# Review of the 100 Most Cited Articles in *Burns* from 2014 to 2024: A Bibliometric Analysis

**DOI:** 10.3390/ebj6020033

**Published:** 2025-06-10

**Authors:** Anna Jolly Neriamparambil, Richard Wong She, Paul Andrew Baker, Lindsay Damkat-Thomas, Joyce Antony

**Affiliations:** 1 National Burn Centre, Department of Plastic and Reconstructive Surgery, Middlemore Hospital, Auckland 2025, New Zealand; anna.jollyneriamparambil@middlemore.co.nz (A.J.N.); richard.wongshe@cmdhb.org.nz (R.W.S.); paul.baker@middlemore.co.nz (P.A.B.); lindsay.damkat-thomas@middlemore.co.nz (L.D.-T.); 2 Princess Alexandra Hospital, Brisbane, QLD 4102, Australia

**Keywords:** burn, surgery, bibliometric, citation, publication

## Abstract

Substantial research interest has been shown over the past ten years in the management of burn injuries. This bibliometric analysis aims to identify and evaluate the most cited articles that have significantly advanced the field of burn injury management. The 100 most cited articles published from January 2014 to September 2024 were collated using the Web of Science database. The full text of each article was meticulously analyzed for descriptive parameters including subject matter, journal of publication, authorship, institutional affiliation, country of origin, and year of publication. The 100 most cited articles had an average of 203 citations, with the most cited article reaching 754 citations and the least cited article cited 105 times. The subjects ranged from enhancing wound care outcomes to metabolic support, fluid management, and infection prevention and management. These articles were distributed across 59 source journals, with 44% of articles having been published in just ten prominent journals. While bibliometric analyses do not accurately gauge scientific merit, this study illuminates the significant contributions to burn management over the past decade and provides valuable insights into research trends in the field.

## 1. Introduction

**Figure 1:**
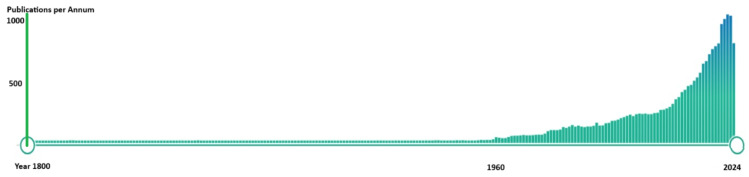
Substantial increase in the number of publications in the field of burn management over the past decade.

Burns can be associated with extensive soft tissue injuries and contribute to significant morbidity and mortality. Research on the management of burns has garnered significant interest over the last decade, as demonstrated in Figure 1. However, identifying the articles that have significantly impacted and advanced the field remains challenging. While the aim of academic research is the generation of robust literature with a high level of evidence, only a minority of publications substantially contribute to the existing body of scientific knowledge.

The importance of articles published in a particular domain is echoed in the quantity of citations received from peers. Citations serve as an acknowledgement by the authors to their colleagues who previously published endeavors in that academic domain. The more times an article is cited, the greater the presumed significance of the article in that particular academic domain. In this context, a bibliometric analysis can be a valuable tool for identifying impactful studies. Therefore, this study focuses on the 100 most cited articles published in the field of burn management from the past decade, aiming to provide insight into significant advancements and key contributions.

## 2. Materials and Methods

**Figure 2:**
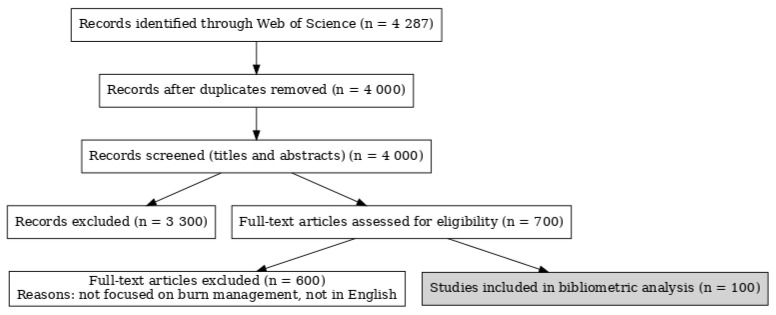
PRISMA flow diagram.

The Web of Science database (Clarivate, Philadelphia, PA, USA) was utilized to perform a comprehensive search and collate articles in the field of burn management published between January 2014 and September 2024 with the highest number of citations. Three authors independently conducted the search using the keyword “burn” to identify all articles in the English language. Articles unrelated to the field of burn management were excluded after a thorough review of the full text of each article. A preliminary list of articles was compiled by combining the results from all three authors. Discrepancies were resolved through a consensus conference of all authors, culminating in the final list of 100 articles relevant to the field of burn management with the highest number of citations. The methodology was based on the approach previously described by Joyce et al. [1]. Each article was systematically analyzed to extract descriptive data, including the topic, the journal and publication year, and the authors, their institutional affiliations, and their country of origin. This process is demonstrated as a PRISMA flow diagram in Figure 2. This study was conducted in accordance with the World Medical Association Declaration of Helsinki and the Good Clinical Practice guidelines.

## 3. Results

In the past decade, the 100 most cited papers in the field of burn management, as demonstrated in Table 1, received an average of 203 citations with a standard deviation of 117 citations. The article with the most citations garnered 754 citations, while the article with the fewest citations had 107 citations.

The top 100 articles on burn management with the most citations in the past ten years were distributed across 59 source journals. Table 2 demonstrates the most prominent ten journals, with impact factors ranging from 2.0 to 98.4, which contributed to 44% of these articles. Further analysis of this list demonstrates that 60% of these articles constituted “review articles” and 66% of these articles were available “open access”. *Elsevier* and *Springer Nature* published 53% of these articles. Funding was a significant feature, with 20% of the articles receiving financial support from the National Institutes of Health (NIH), USA, and a further 20% of the articles receiving financial support from the United States Department of Health and Human Services.

**Figure 3:**
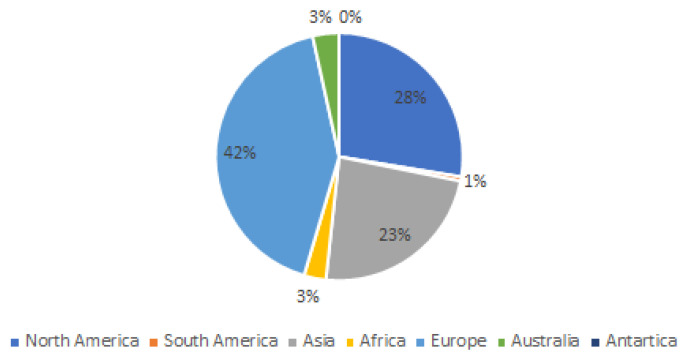
Geographic distribution of authorship (based on continent) that contributed to the list of top 100 articles on burn management with the most citations from 2014 to 2024.

Authorship analysis revealed that seven authors contributed to 29% of the 100 most cited articles on burns management from 2014 to 2024, as demonstrated in Table 3. A gender predilection was noted, with only 23% of the contributions from female authors. Geographically, 42% of the articles originated from European authors, followed by 28% from North American authors, and 23% from Asian authors, as demonstrated in Figure 3. Contributions came from a variety of disciplines, with plastic and reconstructive surgeons and general surgeons making equal contributions to the literature on burn management. Additional contributions were notes from trauma surgeons, pain and rehabilitation physicians, and academics or researchers in basic science and pharmacotherapy.

## 4. Discussion

This bibliometric analysis of burn management identified the 100 most cited articles published over the past decade in English-language literature. Our findings offer valuable insights into significant advancements in this field and highlight key contributions and trends. This list includes major randomized control trials, systematic reviews, and clinical consensus guidelines that contribute an integral knowledge base for the burn surgeon.

Wound care and dressing innovations were a prominent theme that was noted in 17 of the top 100 most cited articles, particularly focussed on rapid healing and prevention of infections [43,76,98]. The most cited article, with 754 citations, is a review of natural and synthetic polymers for wounds and burns dressing published in the *International Journal of Pharmaceutics* [2]. Polysaccharides (alginates, chitin, chitosan, chondroitin, and heparin), proteoglycans and proteins (collagen, eggshell membrane, fibrin, gelatin, keratin, and silk fibroin) are natural polymers used in wounds and burns management because of their biocompatibility, biodegradability and similarity to macromolecules recognized by the body. Synthetic polymers, such as tissue-engineered skin, have been utilized in regenerative medicine for the treatment of severe skin defects or partial-thickness burn injuries.

Critical care management of burns was another prominent theme. The article with the most citations (404) in this domain explored intensive care unit-acquired weakness (ICUAW), a de novo form of muscle weakness, in a substantial number of patients admitted to the ICU with severe burns and other trauma [7]. Other articles in this theme examined the fluid resuscitation of critically ill burns patients, nutrition and metabolism, and prevention and management ICU and hospital acquired concurrent infections [5,62,71]. Another major area of research was the use of virtual reality for pain management. The article with the most citations (200) in this domain was a comprehensive literature review that explored the use of virtual reality as a distraction tool to alleviate pain and distress during medical procedures such as burns debridement and dressing change [36]. Other publications on this theme explored the use of virtual reality for pain and anxiety management in the pediatric burn population [69,70].

This study sheds light on advances since the initial work by Joyce et al. that explored the 100 most influential articles in the field burn management from 1945 to 2013 [1]. Historically, the prominent publications were limited to 27 source journals, including *Annals of Surgery*, *Journal of Trauma*, *Injury, Infection and Critical Care*, *Lancet*, *Burns*, and *New England Journal of Medicine* [1]. In the past decade, impactful work has been distributed across a broader range of journals, reflecting the rise in open-access publishing and the increased digitization of research. Prominent publications spanned 56 source journals, including *Critical Care*, *Burns & Trauma*, and *Burns*, with ongoing contributions from *Lancet* and *Annals of Surgery* to the most prominent burns literature.

The 100 most influential articles on burns from 1945 to 2013 ranged from 104 citations (least cited) to 746 citations (most cited) [1]. This is similar to the 100 most cited articles from 2014 to 2024, which ranged from 105 (least cited) to 754 (most cited). However, this is not similar to bibliometric analyses in other fields, such as hand surgery, during the same period, where citation numbers ranged from 47 (least cited) to 179 (most cited) citations [102].

While journal impact factor is often used as a measure of quality, this does not necessarily determine the journal’s contribution to the list of top 100 articles with the most citations. The impact factor is a measure of the quality of academic journals within an academic domain and is derived by dividing the number of citations from a journal by the number of articles published in that journal over two years [103]. Most of these metrics have significant limitations in that they attribute greater significance to work in academic domains with a larger audience. For example, *Lancet* (impact factor 98.4) appeals to the wider medical audience, while journals such as *Annals of Surgery* (impact factor 10.1) and *Burns & Trauma* (impact factor 6.3) enjoy a smaller, subspecialized readership, yet both have contributed substantially to the literature on burns.

This study has numerous limitations that are inherent to all bibliometric analyses. Citations were utilized as a substitute to comprehend the impact and scientific merit of published articles. Citations may reflect an author’s recognition of the study’s relevance to their research. However, it should be noted that publications from 2023 to 2024 were not included in this list due to insufficient time for the accumulation of citations. Citation bias and self-citation can exaggerate bibliometric results, and non-English articles can have limited visibility. Despite these limitations, this bibliometric analysis offers a snapshot of the impactful research in burn management. It highlights key advancements and provides researchers, surgeons, and allied health professionals with a curated reference of influential studies to inform practice and future investigations [104].

## 5. Conclusions

This bibliometric analysis provides an insightful overview of the 100 most cited articles in burn management from 2014 to 2024. These articles reflect significant advancements and key contributions across diverse areas, including wound care, critical care management, and pain relief innovations. By highlighting the most impactful research, this study serves as a valuable resource for surgeons, researchers, and allied health professionals, offering insights into the foundational work shaping modern burn care. While bibliometric analysis has inherent limitations, it remains a valuable tool for comprehending the evolution of scientific progress in this field.

## Figures and Tables

**Table 1:** The top 100 articles on burn management with the most citations from 2014 to 2024.

Rank	Reference	Citations
1	Mogosanu and Grumezescu [2]	754
2	Jeschke and colleagues [3]	647
3	Rowan and colleagues [4]	562
4	Jault and colleagues [5]	496
5	Finnerty and colleagues [6]	413
6	Hermans and colleagues [7]	404
7	Wang and colleagues [8]	381
8	Kim and colleagues [9]	378
9	Qin and colleagues [10]	364
10	Krausz and colleagues [11]	364
11	Smolle and colleagues [12]	347
12	Portela and colleagues [13]	340
13	Jahromi and colleagues [14]	339
14	Mulcahy and colleagues [15]	329
15	Li and colleagues [16]	328
16	Tavakoli and colleagues [17]	308
17	Lee and colleagues [18]	301
18	Vanhorebeek and colleagues [19]	294
19	Abdullahi and colleagues [20]	292
20	Hobman and colleagues [21]	270
21	Preiser and colleagues [22]	260
22	Bano and colleagues [23]	255
23	Arno and colleagues [24]	232
24	Madaghiele and colleagues [25]	227
25	Balk [26]	223
26	Shpichka and colleagues [27]	220
27	Lachiewicz and colleagues [28]	219
28	Jull and colleagues [29]	218
29	Etulain [30]	212
30	Monstrey and colleagues [31]	212
31	Norbury and colleagues [32]	204
32	Chua and colleagues [33]	203
33	Greenhalgh [34]	202
34	Oudemans-van Straaten and colleagues [35]	201
35	Indovina and colleagues [36]	200
36	Cauwels and colleagues [37]	195
37	Nielson and colleagues [38]	186
38	Peng and colleagues [39]	178
39	Randall and colleagues [40]	178
40	Marshall and colleagues [41]	177
41	Marino and colleagues [42]	173
42	Alven and colleagues [43]	171
43	Augustine and colleagues [44]	170
44	Bassetti and colleagues [45]	170
45	Nisar and colleagues [46]	169
46	Ono and colleagues [47]	169
47	Porter and colleagues [48]	167
48	Shanmuganathan and colleagues [49]	162
49	Ahuja and colleagues [50]	161
50	Rose and colleagues [51]	159
51	Gentile and colleagues [52]	157
52	Hakkarainen and colleagues [53]	156
53	Rosenberg and colleagues [54]	153
54	Morsi and colleagues [55]	153
55	He and colleagues [56]	152
56	Monavarian and colleagues [57]	151
57	Shahrokhi and colleagues [58]	151
58	Ju and colleagues [59]	150
59	Walker and colleagues [60]	147
60	Bahramsoltani and colleagues [61]	143
61	Lewis and colleagues [62]	142
62	Hu and colleagues [63]	142
63	Hadisi and colleagues [64]	140
64	Ter Horst and colleagues [65]	140
65	Bittner and colleagues [66]	138
66	Dai and colleagues [67]	137
67	Shan and colleagues [68]	137
68	Arane and colleagues [69]	135
69	Jeffs and colleagues [70]	135
70	Clark and colleagues [71]	134
71	Jeschke and colleagues [72]	134
72	Huang and colleagues [73]	133
73	El Ayadi and colleagues [74]	130
74	Hampson and colleagues [75]	130
75	Stoica and colleagues [76]	124
76	Hultman and colleagues [77]	124
77	Li and colleagues [78]	123
78	Baradaran-Rafii and colleagues [79]	121
79	Hop and colleagues [80]	121
80	Hassanshahi and colleagues [81]	120
81	Sharma and colleagues [82]	119
82	Gold and colleagues [83]	119
83	Rousseau and colleagues [84]	118
84	Sheikh and colleagues [85]	118
85	Fairbairn and colleagues [86]	118
86	Murray and colleagues [87]	117
87	Oryan and colleagues [88]	117
88	Lantieri and colleagues [89]	116
89	Ramirez and colleagues [90]	115
90	Lambden and colleagues [91]	114
91	Bai and colleagues [92]	114
92	Mehta and colleagues [93]	113
93	Malbrain and colleagues [94]	112
94	Hui and colleagues [95]	112
95	Zhang and colleagues [96]	110
96	Li and colleagues [97]	109
97	Ali and colleagues [98]	109
98	Lee and colleagues [99]	107
99	Guo and colleagues [100]	107
100	Stanojcic and colleagues [101]	105

**Table 2:** Source journals contributing most frequently to the top 100 articles on burn management with the most citations from 2014 to 2024.

Journals	Impact Factor (2023)	Number of Articles
*CRITICAL CARE*	8.8	7
*BURNS & TRAUMA*	6.3	6
*BURNS*	3.2	5
*INTERNATIONAL JOURNAL OF BIOLOGICAL MACROMOLECULES*	7.7	5
*LANCET*	98.4	4
*ADVANCED DRUG DELIVERY REVIEWS*	15.2	4
*INTERNATIONAL JOURNAL OF MOLECULAR SCIENCES*	4.9	4
*STEM CELL RESEARCH & THERAPY*	7.1	3
*JOURNAL OF PLASTIC, RECONSTRUCTIVE AND AESTHETIC SURGERY*	2.0	3
*ANNALS OF SURGERY*	10.1	3

**Table 3:** Authors contributing most frequently to the top 100 articles on burn management with the most citations from 2014 to 2024.

Author	Gender	Department	Institution	Country	Number of Articles
Jeschke, Marc	Male	Burns	Ross Tilley Burn Center; Sunnybrook Research Institute	Canada	8
Herndon, David	Male	Burns	Shriners Hospital; University of Texas	USA	4
Finnerty, Celeste C.	Female	Burns	Shriners Hospital; University of Texas	USA	4
Moiemen, Naiem	Male	Burns, Plastic Surgery	University Hospital Birmingham; Scar Free Foundation Burns Research Centre	UK	4
Van den Berghe, Greet	Female	Intensive Care	University Hospital of Leuven; Hasselt University	Belgium	3
Gibran, Nicole S.	Female	Burns, General Surgery	UW Medicine Regional Burn Center, Harborview Medical Center	USA	3
Chung, Kevin K.	Male	Academic	The Education University of Hong Kong	People’s Republic of China	3

## Data Availability

The authors declare that data will be available upon request.

## Abbreviations

The following abbreviations are used in this manuscript:

NIH	National Institutes of Health
ICUAW	Intensive Care Unit-Acquired Weakness
